# Intrauterine environments and breast cancer risk: meta-analysis and systematic review

**DOI:** 10.1186/bcr1850

**Published:** 2008-01-21

**Authors:** Sue Kyung Park, Daehee Kang, Katherine A McGlynn, Montserrat Garcia-Closas, Yeonju Kim, Keun Young Yoo, Louise A Brinton

**Affiliations:** 1 Department of Preventive Medicine, Seoul National University College of Medicine, Yeongeon-dong, Jongro-gu, Seoul 110-799, Seoul, Republic of Korea; 2 Division of Cancer Epidemiology and Genetics, National Cancer Institute, National Institutes of Health, Executive Blvd, Rockville, Maryland 20892-7234, USA; 3 Cancer Research Institute, Seoul National University, Yeongeon-dong, Jongro-gu, Seoul 110-799, Seoul, Republic of Korea; 4 National Cancer Center, Madu1-Dong, Ilsandong-Gu, Goyang-Si, Gyeonggi-Do 410-769, Republic of Korea

## Abstract

**Introduction:**

Various perinatal factors, including birth weight, birth order, maternal age, gestational age, twin status, and parental smoking, have been postulated to affect breast cancer risk in daughters by altering the hormonal environment of the developing fetal mammary glands. Despite ample biologic plausibility, epidemiologic studies to date have yielded conflicting results. We investigated the associations between perinatal factors and subsequent breast cancer risk through meta-analyses.

**Methods:**

We reviewed breast cancer studies published from January 1966 to February 2007 that included data on birth weight, birth order, maternal age, gestational age, twin status, and maternal or paternal smoking. Meta-analyses using random effect models were employed to summarize the results.

**Results:**

We found that heavier birth weights were associated with increased breast cancer risk, with studies involving five categories of birth weight identifying odds ratios (ORs) of 1.24 (95% confidence interval [CI] 1.04 to 1.48) for 4,000 g or more and 1.15 (95% CI 1.04 to 1.26) for 3,500 g to 3,999 g, relative to a birth weight of 2,500 to 2,599 g. These studies provided no support for a J-shaped relationship of birthweight to risk. Support for an association with birthweight was also derived from studies based on three birth weight categories (OR 1.15 [95% CI 1.01 to 1.31] for ≥4,000 g relative to <3,000 g) and two birth weight categories (OR 1.09 [95% CI 1.02 to 1.18] for ≥3,000 g relative to <3,000 g). Women born to older mothers and twins were also at some increased risk, but the results were heterogeneous across studies and publication years. Birth order, prematurity, and maternal smoking were unrelated to breast cancer risk.

**Conclusion:**

Our findings provide some support for the hypothesis that *in utero *exposures reflective of higher endogenous hormone levels could affect risk for development of breast cancer in adulthood.

## Introduction

Intrauterine environmental exposures to endogenous or exogenous hormones, notably estrogens, may influence the subsequent development of breast cancer in offspring [1]. During pregnancy, levels of circulating estrogens and other hormones with growth-enhancing properties are at least 10 times higher than those in nonpregnant women, with increases seen with advancing gestational age [2-4]. The hypothesis that breast cancer in daughters may be influenced by the intrauterine environment is receiving increased attention [5]. Perinatal factors, including birth weight, birth order, maternal age, gestational age, twin status, and parental smoking, have been postulated as risk factors for breast cancer through altered hormonal influences on the developing fetal mammary glands [1]. Despite ample biologic plausibility, this hypothesis is difficult to evaluate directly [5], and previous epidemiologic studies have reported conflicting results [6,7].

Here we review the epidemiologic studies that have assessed the association between perinatal factors and breast cancer risk in daughters. A meta-analytical approach was applied in order to clarify further the possible role played by the intrauterine environment in the etiology of breast cancer.

## Materials and methods

### Identification of studies

The data retrieved for the systematic review were based on searches of all published papers, letters, abstracts, and review articles on birth weight, birth order, maternal age, gestational age, twin status, and maternal or paternal smoking and breast cancer using the MEDLINE database from January 1966 through February 2007. We used keywords combining text words, with terms for six perinatal factors combined with terms for breast cancer (Table 1). We also manually searched the reference lists of all studies that fulfilled the inclusion criteria for further relevant publications. Articles were included in our systematic review if they fulfilled the following three criteria: the exposure status of at least one of six perinatal risk factors of interest was compared with nonexposure status; the outcome focused on the daughter's breast cancer morbidity or mortality using an epidemiologic study design (case-control design, data linkage study, or cohort study design); and the article was written in English language. We excluded animal studies, investigations focusing on male breast cancer, reviews, and studies that did not provide separate relative risks for breast cancer. We also excluded studies if odds ratios (ORs) or relative risks (RRs) were not specifically provided, raw data were not available for calculation of risks, or the emphasis of analyses was on hazard ratios or standardized incidence ratios.

**Table 1:** Search terms used in systematic review

Subject	Search term
Breast neoplasm	Breast neoplasms, subsequent breast neoplasm, breast neoplasm and daughter
Birth weight	Cirth weight, birthweight, birth size
Birth order	Birth order, birth rank
Maternal age	Maternal age, mother's age, parental age
Gestational age	Gestational age, preterm, prematurity, abruption placenta, pre-eclampsia, eclampsia
Twinship	Twin, twining, multiple births, multiple pregnancy, monozygote twin, dizygote twin,
Parental smoking	Maternal smoking, mother's smoking, paternal smoking, father's smoking, parental smoking
Others	Prenatal factors, perinatal factors, intrauterine environment, intrauterine factor, *In-utero *exposure

### Statistical analyses

For the purposes of meta-analysis, birth weight was classified in three different ways: five categories (<2,500 g, 2,500 to 2,999 g [referent], 3,000 to 3,499 g, 3,500 to 3,999 g, and ≥4,000 g); three categories (<3,000 g [referent], 3,001 to 3,999 g, and ≥4,000 g); and two categories (<3,000 g [or ≤3,000 g; referent] and ≥3,000 g [or >3,000 g]). Birth order was examined using two different categorical schemes: 1 (referent) versus ≥2; and 1 (referent), 2 to 4, and ≥5. Maternal age was classified into three categories: <25 years old (referent), 25 to 29 years old, and ≥30 years old. Gestational age was also analyzed in two ways: ≤36 weeks versus ≥37 weeks (referent); and ≤32 weeks versus ≥33 weeks (referent). To examine twin status, three classification schemes were employed: twin versus singleton (referent); monozygotic twin (or sister twins if zygosity was not reported) versus singleton (referent); and dizygotic twin (or sister-brother twins if zygosity was not reported) versus singleton (referent). Maternal or paternal smoking was considered as follows: no smoking during pregnancy (referent) versus smoking during pregnancy. If the criteria utilized in an article were slightly different from our criteria, then we included the data and described the difference in a footnote.

ORs and 95% confidence intervals (CIs) were recalculated from published frequency tables of individual studies using the Mantel-Haenszel common OR estimate. However, the reported OR (95% CI) was used when the published studies did not provide further details as to the frequencies of the exposure variables. If the manuscript reported the results after performing a stratified analysis, then we re-calculated the crude OR by combining across strata. A random-effects model was used to obtain summary ORs and 95% CIs.

Heterogeneity was assessed by heterogeneity test using Cochran Q statistics [8]. Publication bias was assessed according to the Egger regression asymmetry test, and the Begg and Mazumdar adjusted rank correlation tests [9,10]. The Egger test is a simple linear regression of the natural log of ORs or RRs against its precision (the inverse of its standard error) [10]. The Begg and Mazumdar rank correlation test reports the rank correlation (Kendall's tau) between the standardized effect size and the standard errors of these effects. If asymmetry is caused by publication bias, then we would expect that high standard errors (small studies) would be associated with larger effect sizes (ORs or RRs) or low standard errors (large studies) would be associated with smaller effect sizes [9]. The Begg and Mazumdar test makes fewer assumptions than does the Egger test, but it is insensitive to many types of bias (lower power) that the Egger test is sensitive to [11].

When significant heterogeneity or publication bias was found, we performed subgroup analyses by study design (case-control study versus cohort study), and the source of information (data linkage versus self-report) to assess the impact on between-study variations (heterogeneity). Many of the large-scale studies, especially cohort studies, were published after 2000; thus, we classified publication years into before 2000 versus 2000 or later in order to assess publication biases associated with small study sizes. Few studies focused on either Asians or African-Americans, and so it was not possible to examine ethnicity effects. All statistical analyses were conducted using STATA (Version 8.2 [special edition]; Stata Corp., College Station, TX, USA).

## Results

We identified 34 studies that assessed the association between birth weight and breast cancer risk (Table 2): 19 case-control studies (eight population based, three nested, six record linkage based, and two twin-based) and 15 cohort studies (seven population based and eight record linkage based). Many studies showed positive association between heavier birth weight and breast cancer risk [12-31], and some of the studies observed stronger effects among younger (<45 years) or premenopausal women [14,22,29,30]. In contrast, studies observing no association [32-41] or a negative one [42-45] also have been reported. Additionally, some authors reported a J-shaped relationship between birth weight and breast cancer risk [12,14,15,18,25,33,35,37,45], particularly for early-onset cancers [18].

**Table 2:** Studies assessing the association of birth weight and the risk for breast cancer

Type of study	Ref.	Year	Design	Cases	Controls (or cohort)	Country/place of study	Birthweight (g)	OR (95% CI)	Comments
Case-control studies	[42]	1988	PCC	153	461	USA	1,162–2,948	Referent	Matched analysis; *P *for trend = 0.41
							2,949–3,340	0.65 (0.33–1.26)	
							3,341–4,451	0.76 (0.41–1.43)	
	[12]^a^	1992	LCC	458	1,197	Sweden	<2,500	1.18 (0.60–2.33)	Adjusted for age and birth date
							2,500–2,999	Referent	
							3,000–3,499	1.29 (0.90–1.91)	
							3,500–3,999	1.47 (1.00–2.18)	
							≥ 4,000	1.23 (0.80–2.00)	
	[13]	1996	NCC	550	1,478	USA	<2,500	0.56 (0.34–0.93)	Adjusted for age
							2,500–2,999	0.68 (0.47–0.99)	
							3,000–3,499	0.71 (0.50–0.99)	
							3,500–3,999	0.85 (0.59–1.22)	
							≥ 4,000	Referent	
	[14]	1996	PCC	922	1,194	USA	Age 21–45 years:		Adjusted for age, menopausal status, and maternal smoking; *P *for trend = 0.06 among both groups. The OR (95% CI) for birth weight ≥ 4,000 g among patients with early-onset breast cancer (≤ 30 years old) was 3.3 (1.0–11.0)
							<2,500	1.3 (0.9–2.0)	
							2,500–2,999	Referent	
							3,000–3,499	1.3 (1.0–1.7)	
							3,500–3,999	1.2 (0.8–1.6)	
							≥ 4,000	1.7 (1.1–2.5)	
							Age 50–64 years:		
							<2,500	0.9 (0.5–1.7)	
							2,500–2,999	Referent	
							3,000–3,499	1.1 (0.7–1.7)	
							3,500–3,999	0.8 (0.4–1.3)	
							≥ 4,000	0.6 (0.3–1.1)	
	[32]	1997	NCC	1068	2,027	Sweden	<2,500	0.80 (0.50–1.26)	Adjusted for maternal age, socioeconomic status, parity, and pre-eclampsia or eclampsia, neonatal jaundice, severe prematurity, and twinship
							2,500–2,999	Referent	
							3,000–3,499	1.00 (0.79–1.28)	
							3,500–3,999	0.99 (0.77–1.26)	
							≥ 4,000	1.04 (0.77–1.41)	
	[33]	1998	PCC	510	436	USA	<2,500	1.2 (0.7–2.1)	Crude ORs
							2,500–2,999	Referent	
							3,000–3,499	1.0 (0.7–1.5)	
							3,500–3,999	1.0 (0.7–1.5)	
							≥ 4,000	1.3 (0.7–2.3)	
	[15]	2000	LCC	484	2,870	USA	<1,500	1.59 (0.61–4.11)	Crude ORs
							1,500–2,499	1.33 (0.94–1.90)	
							2,500–3,499	Referent	
							3,500–4,499	1.08 (0.87–1.34)	
							≥ 4,500	3.29 (1.37–7.92)	
	[34]	2001	LTCC	87	87	Sweden	<1,999	Referent	Matched analysis by conditional logistic regression
							2,000–2,499	1.6 (0.6–4.0)	
							2,599–2,999	2.4 (0.9–6.2)	
							≥ 3,000	1.6 (0.4–5.6)	
								(*P *trend = 0.05)	
	[43]	2001	LCC	319	768	USA	<2,500	1.4 (0.55–3.4)	Crude ORs. Higher birth weight (≥ 3,500 g) carried a marginal significantly higher risk for breast cancer (OR 1.76 [95% CI 0.90–3.35]) relative to lower birth weight (<3,500 g)
							2,500–3,750	Referent	
							≥ 3,750	0.9 (0.50–1.6)	
	[16]	2001	LTCC	90	90	Sweden	≤ 2,000	Referent	Crude ORs. Study subjects were women with opposite-sexed pair twins
							2,001–2,500	3.2 (0.8–12.6)	
							2,501–3,000	3.5 (1–13)	
							3,001–3,500	5.8 (1.3–25.7)	
							≥ 3,501	12.1 (1.1–138.8)	
	[35]	2002	PCC	2,088	2,187	USA	<2,500	1.10 (0.90–1.35)	Adjusted for age and residential regions (states)
							2,500–2,999	0.90 (0.70–1.10)	
							3,000–3,499	Referent	
							3,500–3,999	1.07 (0.90–1.30)	
							4,000–4,499	0.89 (0.70–1.14)	
							≥ 4,500	1.18 (0.90–1.51)	
	[44]	2002	PCC	288	350	China	<2,500	0.9 (0.4–2.0)	Adjusted for age income, family history of breast cancer in first-degree relative, history of fibroadenoma, age at menarche, parity, and age at first live birth.
							2,500–2,999	Referent	
							3,000–3,499	1.1 (0.8–1.6)	
							3,500–3,999	0.8 (0.4–1.4)	
							≥ 4,000	0.7 (0.4–1.4)	
	[17]	2002	LCC	373	1,150	USA	<3,090	Referent	Adjusted for parity and age at first birth. *P *for trend = 0.02
							3,090–3,410	1.1 (0.8–1.5)	
							3,420–3,720	1.2 (0.9–1.6)	
							≥ 3,630	1.4 (1.1–1.9)	
	[18]	2003	LCC	881	3,423	Denmark	<2,500	1.66 (1.00–2.51)	Adjusted for mother's marital status, maternal age, and birth order
							2,500–2,999	0.83 (0.60–1.10)	
							3,000–3,499	Referent	
							3,500–3,999	0.98 (0.80–1.17)	
							≥ 4,000	1.25 (1.00–1.55)	
	[19]	2004	NCC	89	238	Sweden	100 g increase	1.06 (1.00–1.12)	Adjusted for gestational age, birth year, and maternal hypertension/proteinuria
	[45]	2004	LCC	2471	9801	USA	<1,500	0.64 (0.40–1.11)	Adjusted for age and maternal age at first birth
							1,500–1,999	1.05 (0.70–1.68)	
							2,000–2,499	1.02 (0.80–1.31)	
							2,500–3,499	Referent	
							3,500–3,999	0.97 (0.90–1.08)	
							4,000–4,499	0.93 (0.80–1.11)	
							≥ 4,500	0.69 (0.40–1.09)	
	[36]	2004	PCC	196	167	USA	All subjects:		Adjusted for age, race and sampling fractions, body mass index, household income, and maternal age. Tertiles are race specific with cutpoints derived from controls. White women: <3,062, 3,062–3,458, >3,458 g; black women: <3,146, 3,146–3,488, >3,488 g. Restricted data using birth weight measured in pounds and ounces and participant delivered in a medical facility by a physician
							Lower tertile	1.0 (0.6–1.7)	
							Central tertile	Referent	
							Upper tertile	0.7 (0.4–1.2)	
							White, restricted		
							data:		
							Lower tertile	1.1 (0.5–2.4)	
							Central tertile	Referent	
							Upper tertile	1.4 (0.6–2.0)	
	[20]	2006	PCC	2,386	2,502	Poland	<2,500	Referent	Adjusted for: age, education, age at menarche, menopausal status and age at menopause, age at first full-term pregnancy, number of full-term pregnancies, family history of breast cancer among first-degree relatives, mammography screening, and current body mass index. Lower birth weight (<2,500 g) carries greater risk than birth weight of 2,500–4,000 g among women under 45 years old
							2,500–4,000	1.22 (0.92–1.62)	
							>4,000	1.54 (1.08–2.19)	
								(*p*-trend = 0.01)	
	[37]	2006	PCC	1,166	2,105	USA	<2,495	1.19 (0.85–1.66)	Adjusted for age (years), education (years), race, body mass index, history of breast benign disease, family history of breast cancer, lactation (months), age at menarche (years), age at first full-term pregnancy (years), age at menopause (years), parity
							2,495–3,130	Referent	
							3,131–3,855	0.97 (0.75–1.25)	
							>3,855	1.03 (0.74–1.44)	

Cohort studies	[21]	1999	LCohort	57	152,590	Sweden	<2,500	Referent	Standardization for sex, age, and age-specific incidence rate
							2,500–3,999	1.3 (0.6–2.4)	
							4,000–4,499	1.2 (0.0–6.7)	
							≥ 4,500	1.3 (0.7–2.3)	
	[22]	2000	Cohort	37	2,221	UK	All ages		Adjusted for age. *P *for trend = 0.03 among premenopausal women
							<3,000	Referent	
							3,000–3,499	1.05 (0.41–2.71)	
							3,500–3,999	1.76 (0.72–4.33)	
							≥ 4,000	2.02 (0.59–6.90)	
							Premenopausal ages		
							<3,000	Referent	
							3,000–3,499	1.99 (0.40–9.86)	
							3,500–3,999	3.26 (0.69–15.36)	
							≥ 4,000	5.65 (0.95–33.84)	
	[38]	2001	LCohort	177	3,447	Sweden	≤ 2,000	Referent	Crude hazard ratios
							2,001–2,500	1.4 (0.6–3.4)	
							2,501–3,000	1.9 (0.8–4.3)	
							3,001–3,500	1.5 (0.6–3.5)	
							≥ 3,501	1.9 (0.7–5.0)	
	[39]	2001	Cohort	62	1260	Sweden	≤ 3,000	Referent	Singleton only; adjusted for gestational age and cohort membership
							3,010–3,349	1.16 (0.47–2.87)	
							3,350–3,590	1.65 (0.71–3.86)	
							3,600–3,960	1.58 (0.67–3.72)	
							≥ 4,000	1.57 (0.67–3.64)	
	[23]	2003	LCohort	63	5,352	Sweden	<3,000	Referent	Crude ORs;*P *for trend = 0.01
							3,000–3,499	1.46 (0.60–3.43)	
							3,500–3,999	2.09 (0.90–4.85)	
							≥ 4,000	2.78 (1.10–7.15)	
	[24]	2003	LCohort	2,334	106,504	Denmark	1,000 g increase	9 (0.02–17)%	Adjusted for age and calendar period. Additional adjustment for parity and age at first birth did not indicate confounding
	[25]	2003	LCohort	39	1483	Sweden	500–1,999	1.14 (0.70–1.85)	Standardized incidence ratio (expected/observed)
							2,000–2,999	0.71 (0.40–1.15)	
							≥ 3,000	2.55 (1.03–5.25)	
	[26]^a^	2004	LCohort	2,074	91,601	Denmark	Median of each quintile		Adjusted for age and calendar period. No change in estimates when additionally adjusted for parity and age at first birth
							2.5	Referent	
							3.0	0.98 (0.85–1.13)	
							3.4	1.06 (0.93–1.20)	
							3.6	1.05 (0.87–1.27)	
							4.0	1.17 (1.02–1.33)	
	[27]	2004	Cohort	59	2,176	UK	<3,000	Referent	Adjusted for age; *P *for trend = 0.03
							3,000–3,499	1.37 (0.34–5.47)	
							3,500–3,999	2.18 (0.58–8.21)	
							≥ 4,000	5.03 (1.13–22.47)	
	[28]	2005	LCohort	311	16,011	USA	<3,040	Referent	Adjusted for year of birth
							3,040–3,310	1.4 (1.0–2.1)	
							3,320–3,550	1.0 (0.6–1.5)	
							3,560–3,830	1.3 (0.9–1.9)	
							≥ 3,840	1.5 (1.0–2.2)	
	[29]^a^	2005	LCohort	367	5,346	Sweden	<50 years		
							<3,000	Referent	
							3,000–3,499	1.81 (0.77–4.26)	
							3,500–3,999	2.66 (1.09–6.46)	
							≥ 4,000	4.00 (1.49–10.72)	
							≥ 50 years		
							<3,000	Referent	
							3,000–3,499	0.86 (0.62–1.19)	
							3,500–3,999	1.06 (1.20–3.34)	
							≥ 4,000	0.91 (0.57–1.46)	
	[40]	2006	Cohort	97	5,847	USA	<3,000	0.98 (0.61–1.60)	Adjusted for age
							3,000–3,499	Referent	
							≥ 3,500	1.09 (0.66–1.80)	
	[30]	2006	Cohort	3,140	91,601	USA	Premenopause		Adjusted for age: *P *for trend = 0.019
							<2,495	0.69 (0.50–0.94)	
							2,495–3,130	0.79 (0.64–0.97)	
							3,131–3,810	0.76 (0.63–0.93)	
							>3,810	Referent	
							Postmenopause:		Adjusted for age: *P *for trend = 0.99
							<2,495	1.04 (0.88–1.23)	
							2,495–3,130	1.00 (0.87–1.14)	
							3,131–3,855	1.05 (0.93–1.20)	
							>3,855	Referent	
	[31]	2006	Cohort	209	1,024	USA	<2,500	0.9 (0.5–1.6)	Hazard ratio; adjusted for age at diagnosis, diagnosis year, stage at diagnosis, and birth order, with exception of birth order, which is adjusted for maternal age
							2,500–3,999	Referent	
							≥ 4,000	1.8 (1.0–3.1)	
								(*P *trend = 0.1)	
	[41]	2007	Cohort	657	38,566	Sweden	<2,500	0.65 (0.43–0.99)	Adjusted for adult body mass index
							2,500–3,000	1.04 (0.86–1.25)	
							>3,000	Referent	

Cohort, cohort study; LCC, case-control study with linkage with population and cancer registry data; LCohort, cohort study with linkage with population and cancer registry data; LTCCS, twin case-control study by using linkage with birth and cancer registry data; NCC, nested case-control study in cohort; PCC, population-based case-control study. ^a^The numbers of cases and controls were not shown in the original article.

**Figure 1:**
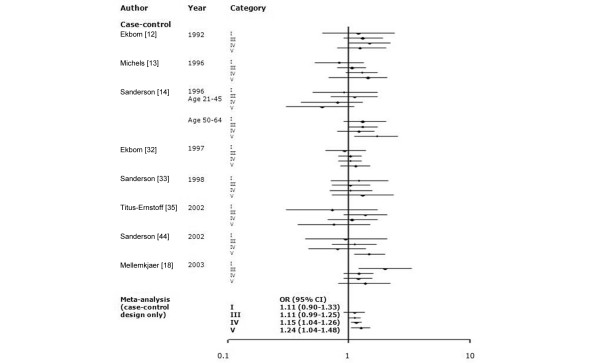
Meta-analysis of the association between birth weight (five categories) and risk for breast cancer. The tests for homogeneity and for publication bias in the studies analyzed are as follows. Category I (birth weight <2,500 g) versus reference: Q = 9.66 (8 degrees of freedom), *P *= 0.29; Begg test, *P *= 0.75; Egger test, *P *= 0.66. Category II (2,500 to 2,999 g) is the reference. Category III (3,000 to 3,499 g) versus reference: Q = 6.53 (8 degrees of freedom), *P *= 0.59; Begg test, *P *= 0.25; Egger test, *P *= 0.46. Category IV (3,500 to 3,999 g) versus reference: Q = 4.17 (8 degrees of freedom), *P *= 0.84; Begg test, *P *= 0.60; Egger test, *P *= 0.93. Category V (≥4,000 g) versus reference: Q = 11.18 (8 degrees of freedom), *P *= 0.19; Begg test, *P *= 0.25; Egger test, *P *= 0.30. ^1^We used adjusted odds ratios (ORs) for meta-analysis because the numbers of cases and controls were not represented in the original article. CI, confidence interval.

**Figure 2:**
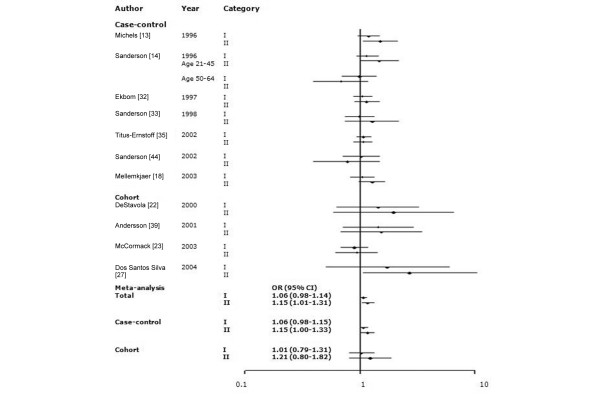
Meta-analysis of the association between birth weight (three categories) and risk for breast cancer. The tests for homogeneity and for publication bias in the studies analyzed are as follows. Category I (birth weight 3,000 to 3,999 g) versus reference (<3,000 g): Q = 4.97 (11 degrees of freedom), *P *= 0.93; Begg test, *P *= 0.54; Egger test, *P *= 0.27. Category II (≥4,000 g) versus reference: Q = 13.44 (11 degrees of freedom), *P *= 0.27; Begg test, *P *= 0.54; Egger test, *P *= 0.53. CI, confidence interval; OR, odds ratio.

**Figure 3:**
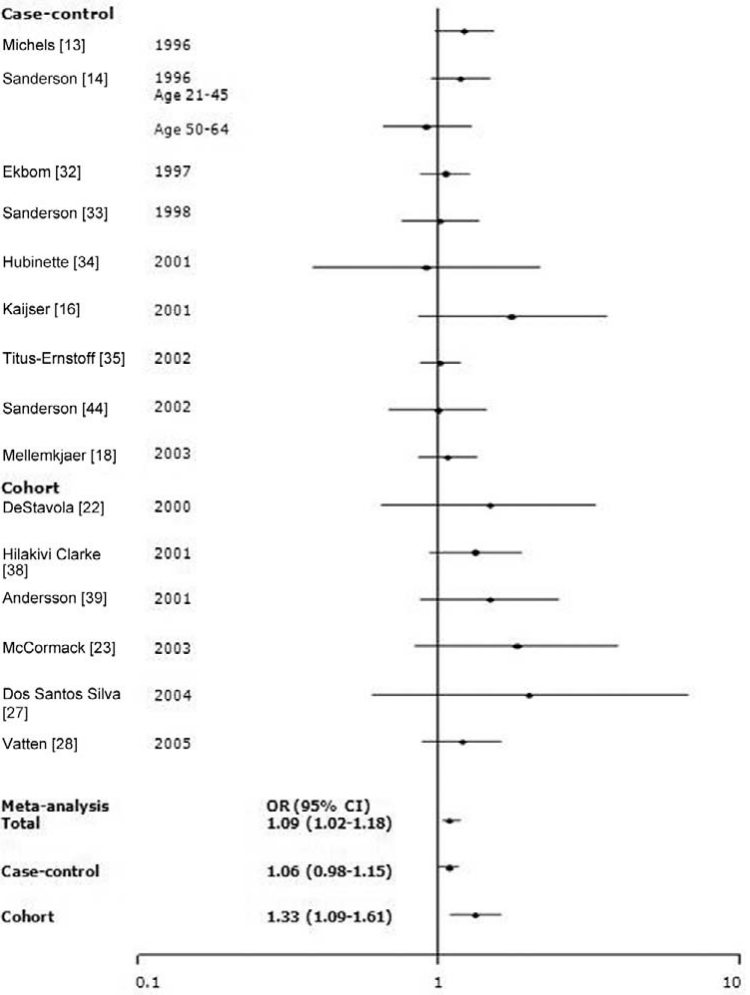
Meta-analysis of the association between birth weight (two categories) and risk for breast cancer. The tests for homogeneity and for publication bias in the studies analyzed are as folows. Reference (<3,000 g [or ≤3,000 g]) versus ≥3,000 g (or >3,000 g): Q = 11.57 (15 degrees of freedom), *P *= 0.93; Begg test, *P *= 0.15; Egger test, *P *= 0.50. CI, confidence interval; OR, odds ratio.

Among 34 studies of birth weight and breast cancer, we selected studies that employed the same categories of birth weight. To evaluate whether a J-shaped relationship existed, we grouped birth weight into more than three categories. The findings of meta-analysis of eight studies that utilized five categories of birth weight (<2,500, 2,500 to 2,999, 3,000 to 3,499, 3,500 to 3,999, and ≥4,000 g) and 11 studies that used three categories (<3,000, 3,000 to 3,999, and ≥4,000 g) are shown in Figures 1 and 2. To include more studies, we also categorized birthweights as <3,000 g (or ≤3,000 g) and ≥3,000 g (or >3,000 g; Figure 3). Sixteen studies among all 34 studies were included in the meta-analyses for birth weight and breast cancer: seven studies [13,14,18,32,33,35,44] were included in the all three meta-analyses; four studies [22,23,27,39] were included in the two of the three meta-analyses; and five studies were included in only one meta-analysis [12,16,28,34,38]. There was no significant heterogeneity across studies (*P_*Q test > 0.05 for all categories). In the five-category meta-analysis, ORs were 1.11 (95% CI 0.90 to 1.33) for birth weight <2,500 g, 1.11 (0.99 to 1.25) for 3,000 to 3,499 g, 1.15 (1.04 to 1.26) for 3,500 to 3,999 g, and 1.24 (1.04 to 1.48) for ≥4,000 g relative to the referent category of 2,500 to 2,999 g. In the three-category meta-analysis, ORs were 1.06 (95% CI 0.98 to 1.14) for 3,000 to 3,999 g and 1.15 (1.01 to 1.31) for ≥4,000 g relative to the referent category of <3,000 g. In the two-category meta-analysis, ORs were 1.09 (95% CI 1.02 to 1.18) for the category of >3,000 g (or ≥3,000 g) relative to the referent category of ≤3,000 g (or <3,000 g).

We identified 17 studies (15 case-control and two cohort) that assessed the association between birth order and breast cancer risk (Table 3). Eight of the studies reported an inverse relationship [14,20,31,35,36,42,46,47]. Some studies found significantly lower risks for second or later born children versus first-born children [31,46]. Some studies found significantly or marginally significantly reduced risk among women whose birth had been preceded by the birth of at least five siblings [20,35]. Other several studies noted an increased risk associated with higher birth order [15,37,48,49], whereas some studies failed to observe such an association [12,18,32,50]. One study did not supply the estimated risk but describe the *P *value by the mean difference of birth order [51].

**Table 3:** Studies assessing the association of birth order and the risk of breast cancer

Type of study	Ref.	Year	Design	Cases	Controls (or cohort)	Country/place of study	Birth order	OR (95% CI)	Comments
Case-control studies	[51]	1967	LCC-D	229	229	USA	1	-	The authors measured the mean value of birth weight instead of providing ORs (95% CIs). The mean difference between cases and matched controls was not significant (*P *> 0.2). They provided the frequency of each case and control in the tables and we calculated crude ORs
							2		
							3		
							4		
							5		
							6		
							≥ 7		
	[50]^a^	1980	MCC	4339	12,760	USA, Japan, Slovenia, Athens, Taipei	1	Referent	The risks (point estimates) only by birth order were shown in the figure in the original article.
							2	0.93	
							3	1.08	
							4	0.99	
							5	1.05	
							6	1.07	
							7	1.18	
							≥ 8	1.02	
	[42]	1988	PCC	153	461	USA	1	Referent	*P *for trend = 0.16
							2	0.92 (0.55–1.54)	
							3	0.98 (0.58–1.72)	
							4	0.69 (0.36–1.32)	
							≥ 5	1.03 (0.60–1.79)	
	[46]	1991	MCC	927	2,616	USA/Wales/Japan	All ages		Adjusted for age, study center, parity, age at first birth, age at menarche, height, body mass index, maternal age at birth, and menopausal status
							1	Referent	
							2	0.91 (0.73–1.02)	
							3	1.11 (0.87–1.27)	
							≥ 4	1.09 (0.81–1.18)	
							Premenopausal		
							1	Referent	
							≥ 2	0.76 (0.60–0.96)	
	[12]	1992	LCC	458	1,197	Sweden	1	Referent	Adjusted for age and birth date
							≥ 2	1.00 (0.76–1.32)	
	[47]	1994	PCC	2,414	9,138	USA	1	Referent	Adjusted for age at first birth and number of children
							2	0.90 (0.78–1.03)	
							3	0.98 (0.84–1.14)	
							4	0.86 (0.73–1.02)	
							5	0.93 (0.78–1.11)	
							6	1.02 (0.84–1.23)	
							7	0.91 (0.73–1.14)	
							≥ 8	0.88 (0.75–1.04)	
	[14]	1996	PCC	1,129	1,393	USA	1	Referent	Adjusted for age, menopausal status, and maternal smoking; *P *for trend = 0.06 among both groups
							2	1.0 (0.7–1.4)	
							≥ 3	0.8 (0.6–1.1)	
	[32]	1997	NCC	1,068	2,727	Sweden	1	Referent	Adjusted for maternal age, socioeconomic status, parity, and preeclampsia or eclampsia, neonatal jaundice, severe prematurity, and twinship
							2	1.01 (0.83–1.22)	
							≥ 3	1.01 (0.81–1.26)	
	[15]	2000	LCC	481	2,863	USA	1	1.07 (0.84–1.35)	Crude ORs
							2–3	Referent	
							4–5	1.06 (0.81–1.38)	
							≥ 6	1.50 (1.06–2.13)	
	[35]	2002	PCC	1,555	1,539	USA	1	Referent	Adjusted for age and residential regions (states)
							2	1.07 (0.88–1.30)	
							3	1.07 (0.85–1.35)	
							4	1.01 (0.77–1.31)	
							5	0.66 (0.48–0.92)	
							≥ 6	0.81 (0.62–1.08)	
	[18]	2003	LCC	881	3,423	Denmark	1	Referent	Adjusted for mother's marital status, maternal age, and birth order
							≥ 2	1.01 (0.83–1.12)	
	[36]	2004	PCC	854	785	USA	All subjects		Adjusted for age, race and sampling fractions, body mass index, hosehold income, maternal age
							1	Referent	
							2–4	0.9 (0.7–1.1)	
							≥ 5	1.0 (0.8–1.3)	
							Born ≥ 1948		
							1	Referent	
							2–4	0.9 (0.6–1.4)	
							≥ 5	0.6 (0.3–1.3)	
	[48]^a^	2005	MCC	24	34	Nigeria	≤ 3	Referent	Crude ORs
							≥ 4	1.50 (0.25–8.98)	
	[20]	2005	PCC	1642	1,713	Poland	1	Referent	Adjusted for age, education, age at menarche, menopausal status and age at menopause, age at first full-term pregnancy, number of full-term pregnancies, family history of breast cancer among first-degree relatives, mammography screening, and current body mass index
							2	1.07 (0.91–1.24)	
							3–5	0.99 (0.85–1.15)	
							≥ 6	0.81 (0.61–1.06)	
								*P *for trend = 0.81	
	[37]	2006	PCC	1,166	2,105	USA	1	Referent	Adjusted for age (years), education (years), race, body mass index, history of breast benign disease, family history of breast cancer, lactation (months), age at menarche (years), age at first full-term pregnancy (years), age at menopause (years), parity
							≥ 2	1.27 (0.88–1.85)	
Cohort studies	[31]	2006	Cohort	209	1,024	USA	1	Referent	Hazard ratio for breast cancer mortality: adjusted for age at diagnosis, diagnosis year, stage at diagnosis, and birth order, with exception of birth order, which is adjusted for maternal age
							2	0.2 (0.2–0.3)	
							≥ 3	0.2 (0.2–0.3)	
								*P *for trend < 0.01	
	[49]^a^	2001	Cohort	-	-	Sweden	Continuous scale	1.05 (1.01–1.10)	

^a^We did not include these studies in the meta-analysis because they employed different categories or a continuous scale, or they did not provide the numbers of cases and controls in the original article. Cohort, cohort study; LCC, case-control study with linkage with population and cancer registry data; LCC-D, case-control study with linkage with population and cancer death certification data; MCC, multicenter case-control study; NCC, nested case-control study in cohort; PCC, population-based case-control study.

**Figure 4:**
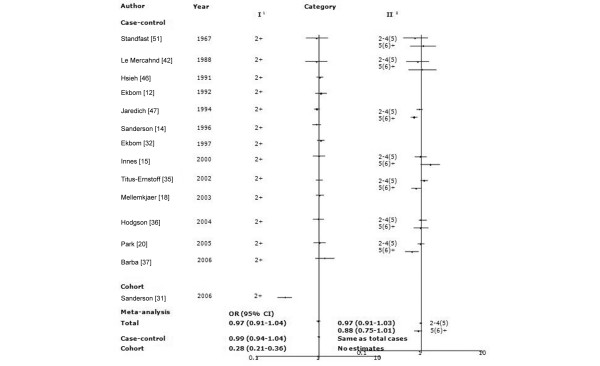
Meta-analysis of the association between birth order and risk for breast cancer. The tests for homogeneity and for publication bias in the studies analyzed are as follows. Category I (birth order 2+) versus reference (birth order 1): Q = 87.79 (13 degrees of freedom), *P *< 0.01; Begg test, *P *= 0.44; Egger test, *P *= 0.46. Category II (birth order 5+ and 2 to 4) versus reference: Q = 4.56 (6 degrees of freedom), *P *= 0.60; Begg test, *P *= 0.37; Egger test, *P *= 0.44. Category II (birth order ≥6, 2 to 5) versus reference: Q = 14.42 (6 degrees of freedom), *P *= 0.60; Begg test, *P *= 0.37; Egger test, *P *= 0.44. ^1^Category I of birth order was 2+ vs 1. ^2^Category II of birth order was composed of two conditions: 5+ and 2 to 4; and ≥6 and 2 to 5 vs 1. ^3^We used adjusted odds ratios (ORs) for meta-analysis because the numbers of cases and controls were not represented in the original article. CI, confidence interval.

For the meta-analysis, we included 14 studies (13 case-control studies and one cohort) that used two birth order categories: 1 (referent) and ≥2. There was significant heterogeneity across all studies (*P_*Q test < 0.01), although there was no significant heterogeneity across the case-control studies (*P_*Q test = 0.90). As shown in Figure 4, there was no difference in risk according to birth order across all studies (OR 0.97 [95% CI 0.91 to 1.04]) or within the case control studies (OR 0.99 [95% CI 0.94 to 1.04]). We calculated the crude odds ratio from the cohort study [31], and the result was very different from the summary OR (calculated crude OR 0.28 [95% CI 0.21 to 0.36]). The results of all case-control studies were near null, whereas the cohort study found a significant risk reduction in birth orders of 2 or greater. We also examined the seven studies that classified individuals according to three birth order levels (1 [referent], 2 to 4, ≥5; Figure. 4). There was significant heterogeneity across studies (all of which were case-control studies) for the highest birth order category (*P_*Q test = 0.03) Women with a birth order of ≥5 were at nonsignificantly reduced risk compared with first-born women (OR 0.88 [95% CI 0.75–1.01]). There was no difference in risk for women of birth orders 2 to 4 (OR 0.97 [95% CI 0.91–1.03]).

We identified 28 studies (22 case-control and six cohort) that assessed the association between maternal age and breast cancer risk (Table 4). Seven studies observed modestly increased risks for daughters born to older mothers [15,31,32,36,42,46,52]. A pattern of slight decrease after modest increase in risk was found in five other studies [50,53-56]. Fourteen studies, however, no association was observed [12,14,18,20,35,37,38,47,49,57-60]. Two studies did not estimate the risks [51,61].

**Table 4:** Studies assessing the association of maternal age with risk for breast cancer

	Ref.	Year	Design	Cases	Controls (or cohort)	Country/place of study	Maternal age (years)	OR (95% CI)	Comments
Case-control studies	[51]	1967	LCC-D	229	229	USA	≤ 19		Mean maternal age among cases was higher than that among controls (*P *< 0.005). The frequency of each case and control were shown in the tables provided and we calculated crude ORs
							20–24		
							25–29		
							30–34		
							35–39		
							≥ 40		
	[61]^a^	1974	PCC	308	308	USA			Matched analysis; the mean maternal age was 27.3 years among cases and 26.3 years among controls (*P *< 0.01)
	[50]^b^	1980	MCC	4339	12760	USA, Japan, Slovenia, Athens, Taipei	≤ 19	Referent	Authors showed point estimates of ORs without 95% CIs. The frequencies for each case and control were given in the tables provided and we calculated crude ORs
							20–24	1.05	
							25–29	1.22	
							30–34	1.19	
							35–39	1.31	
							≥ 40	1.18	
	[53]^a^	1984	MCC	1,176	1,176	England	≤ 20	Referent	Adjusted for age, social class, family history of breast cancer, age at first-term birth, past history of benign breast disease, age at menarche, menopausal status, cigarette smoking, and oral contraceptive use
							21–25	1.41 (0.92–2.18)	
							26–30	1.19 (0.78–1.81)	
							31–35	1.29 (0.83–1.98)	
							≥ 36	1.19 (0.68–1.67)	
	[42]^a^	1988	PCC	153	461	USA	All women		Matched analysis
							15–22	1.18 (0.71–1.97)	
							23–26	Referent	
							27–30	1.22 (0.71–2.10)	
							31–46	1.66 (0.99–2.78)	
								*P *for trend = 0.67	
							Younger women		
							15–23	1.39 (0.65–2.95)	
							24–28	Referent	
							29–46	2.21 (1.02–4.80)	
								*P *for trend = 0.08	
	[52]^a^	1989	PCC	801	1,573	USA	Continuous	1.24 (1.09–1.41)	Crude OR
	[54]	1990	PCC	2,291	3,144	USA	≤ 19	Referent	Adjusted for age and parity, age at first pregnancy, total duration of breast feeding, race, age at menarche, menopausal status, body mass index, family history of breast cancer, and breast biopsy
							20–24	0.95 (0.77–1.16)	
							25–29	1.13 (0.92–1.38)	
							30–34	1.16 (0.93–1.45)	
							35–39	1.46 (1.10–1.93)	
							≥ 40	1.20 (0.79–1.83)	
	[55]	1991	PCC	1761	1,116,553 person-years	USA	≤ 19	Referent	Crude ORs
							20–24	1.02 (0.82–1.46)	
							25–29	1.12 (1.04–1.38)	
							30–34	1.16 (0.93–1.44)	
							35–39	1.17 (0.92–1.48)	
							≥ 40	1.08 (0.80–1.46)	
	[46]^a^	1991	MCC	927	2616	USA, Wales, Japan	Each 5-yrs	1.06 (1.01–1.10)	Adjusted for age, study center, parity, age at first birth, age at menarche, height, BMI, maternal age at birth, and menopausal status
	[12]^b^	1992	LCC	458	1,197	Sweden	Each 5-year band	1.01 (0.92–1.12)	Adjusted for age and birth date. The authors estimated breast cancer risk according to each 5-year band of maternal age. The frequency of each case and control were given in the tables provided and we calculated crude ORs
	[47]	1994	PCC	2,412	9,138	USA	≤ 19	Referent	Adjusted for age at first birth and number of children
							20–24	1.05 (0.85–1.30)	
							25–29	1.10 (0.89–1.37)	
							30–34	1.10 (0.88–1.37)	
							35–39	1.09 (0.87–1.37)	
							≥ 40	0.99 (0.76–1.28)	
	[14]	1996	PCC	1,934	2,161	USA	≤ 24	Referent	Adjusted for age, menopausal status, and maternal smoking
							25–29	1.0 (0.8–1.2)	
							30–34	0.9 (0.6–1.1)	
							≥ 35	1.0 (0.7–1.5)	
	[57]	1997	PCC	1,253	1,121	USA	≤ 19	Referent	Adjusted for age, menopausal status, age at menarche, parity, age at first birth, body mass index, past history of benign breast disease, and recent alcohol intake
							20–24	0.84 (0.62–1.14)	
							25–29	1.02 (0.76–1.37)	
							30–34	0.93 (0.68–1.28)	
							35–39	1.16 (0.82–1.65)	
							≥ 40	0.92 (0.62–1.37)	
	[58]	1997	PCC	2,106	1,926	USA	≤ 19	Referent	Adjusted for age, study site, family history of breast cancer, breast biopsy, a combination variable including number of full-term births and age at first full-term pregnancy, age at menarche, menopausal status, body mass index, average lifetime alcohol consumption, and the number of mammograms
							20–24	0.96 (0.7–1.2)	
							25–29	0.96 (0.7–1.2)	
							30–34	0.91 (0.7–1.2)	
							≥ 35	0.93 (0.7–1.3)	
	[32]^a^	1997	NCC	1,067	2,725	Sweden	Each 5-year band	1.06 (0.99–1.14)	Adjusted for maternal age, socioeconomic status, parity, and pre-eclampsia or eclampsia, neonatal jaundice, severe prematurity, and twinship
	[15]	2000	LCC	481	2863	USA	≤ 19	1.19 (0.83–1.72)	Crude ORs
							20–24	Referent	
							25–29	1.26 (0.97–1.64)	
							30–34	1.38 (1.04–1.84)	
							≥ 35	1.70 (1.23–2.35)	
	[35]	2002	PCC	1,555	1,539	USA	≤ 19	1.02 (0.75–1.39)	Adjusted for age and state
							20–24	0.98 (0.81–1.18)	
							25–29	Referent	
							30–34	1.15 (0.93–1.42)	
							35–39	1.22 (0.94–1.58)	
							≥ 40	1.27 (0.90–1.69)	
	[18]	2003	LCC	881	3,423	Denmark	≤ 24	Referent	Adjusted for mother's marital status, maternal age, and birth order
							25–29	1.08 (0.88–1.32)	
							≥ 30	1.11 (0.90–1.36)	
	[36]^a^	2004	PCC	854	785	USA	≤ 18	1.8 (0.9–3.4)	
							19–22	Referent	Adjusted for age, race and sampling fractions; tertiles are race specific with cutpoints derived from controls
							23–27	3.0 (1.8–5.0)	
							≥ 28	2.5 (1.6–4.0)	
	[56]	2005	MCC	1,060	1,060	Korea	≤ 24	Referent	Adjusted for age, family history of breast cancer in first-or second-degree relatives, menopausal status, and lifetime estrogen exposure duration
							25–29	1.2 (0.93–1.47)	
							30–34	1.4 (1.12–1.83)	
							≥ 35	1.1 (0.83–1.37)	
	[20]	2006	PCC	1,642	1,713	Poland	≤ 19	Referent	Adjusted for: age, education, age at menarche, menopausal status and age at menopause, age at first full-term pregnancy, number of full-term pregnancies, family history of breast cancer among first-degree relatives, mammography screening, and current body mass index
							20–24	1.02 (0.75–1.39)	
							25–29	1.07 (0.79–1.46)	
							35–39	1.16 (0.84–1.60)	
							≥ 35	0.91 (0.66–1.27)	
								*P *for trend = 0.76	
	[37]^a^	2006	PCC	1,166	2,105	USA	≤ 24	Referent	Adjusted for: age, education, race, body mass index, history of breast benign disease, family history of breast cancer, lactation, age at menarche, age at first full-term pregnancy, age at menopause, and parity
							25–35	0.87 (0.67–1.13)	
							>35	0.87 (0.59–1.27)	
Cohort studies	[59]	1995	Cohort	149	75,237	USA	≤ 24	Referent	Adjusted for age, education, menopausal status, parity, body mass index, height, smoking, and alcohol drinking
							25–29	1.3 (0.8–2.0)	
							30–34	1.4 (0.9–2.1)	
							≥ 35	1.2 (0.7–2.0)	
	[60]	1995	Cohort	1,967	384,769	Sweden	≤ 19	Referent	Breast cancer mortality; adjusted for age
							20–24	0.99 (0.82–1.21)	
							25–29	1.00 (0.82–1.22)	
							30–34	0.97 (0.79–1.18)	
							35–39	1.04 (0.84–1.29)	
							40–44	0.93 (0.71–1.21)	
							≥ 45	1.39 (0.91–2.13)	
	[49]^a^	2001	Cohort			Sweden	Continuous scale	1.07 (0.91–1.27)	Adjusted for spouse age, year of diagnosis, and birth order
	[38]^a^	2001	Cohort	177	3,447	Filand	Continuous scale	-	No association
	[31]	2006	Cohort	249	1,024	USA	≤ 24	Referent	Hazard ratio; adjusted for age at diagnosis, diagnosis year, stage at diagnosis, and birth order, with exception of birth order, which is adjusted for maternal age
							25–29	1.2 (0.9–1.7)	
							30–34	1.4 (0.9–1.9)	
							≥ 35	1.7 (1.1–2.8)	
								*P for *trend = 0.03	

^a^We did not include these studies in the meta-analysis because they employed different categories or a continuous scale, or they did not provide the numbers of cases and controls in the original article^b^We included this study in the meta-analysis because we calculated the crude OR using the number of subjects represented the original article. Cohort, cohort study; LCC, case-control study with linkage with population and cancer registry data; LCC-D, case-control study with linkage with population and cancer death certification data; MCC, multicenter case-control study; NCC, nested case-control study in cohort; PCC, population-based case-control study.

**Figure 5:**
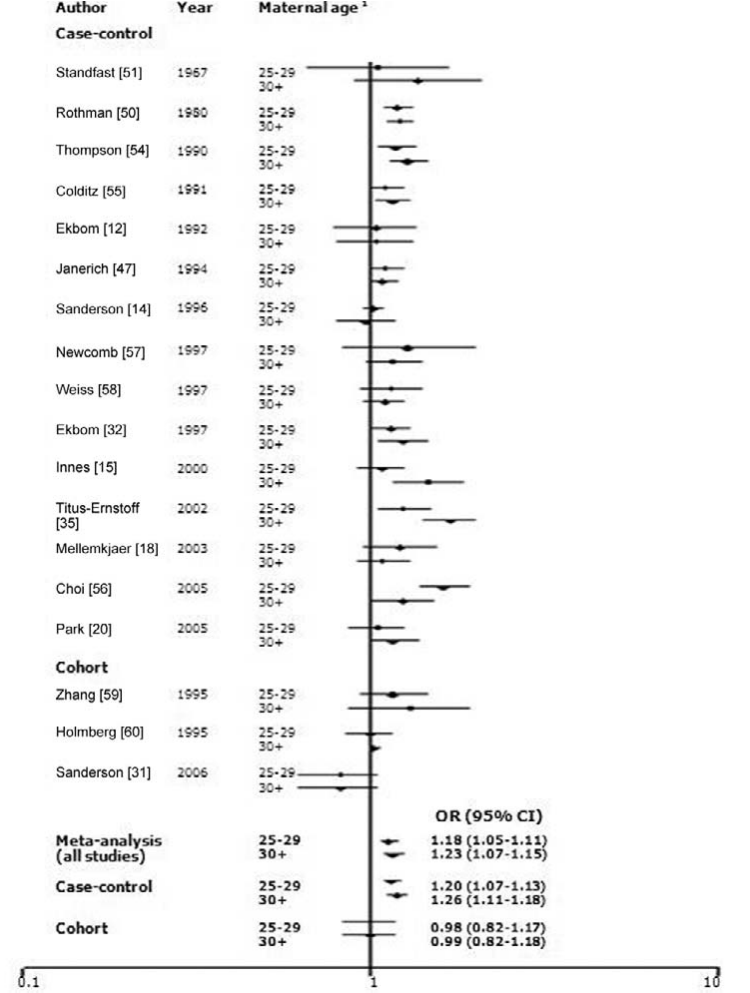
Meta-analysis for the association between maternal age and the risk of breast cancer. The tests for homogeneity and for publication bias in the studies analyzed are as follows. Maternal age 25 to 29 years: Q = 39.40 (17 degrees of freedom), *P *< 0.01; Begg test, *P *= 0.85; Egger test, *P *= 0.38, Maternal age 30+ years: Q = 67.34 (17 degrees of freedom), *P *< 0.01; Begg test, *P *= 0.88; Egger test, *P *= 0.07. ^1^The reference for maternal age is ≤24 years old. CI, confidence interval; OR, odds ratio.

In our meta-analyses, we included the 18 studies that reported categorical data and examined three age categories (≤24 [referent], 25 to 29, and ≥30 years; Figure 5). There was, however, significant study heterogeneity (*P*_Q test < 0.01 for 25 to 29 years and for ≥30 years). Heterogeneity was also present across case-control studies and studies published after 2000 (*P*_Q test < 0.01). The ORs (95% CI) were 1.18 (1.05 to 1.11) for 25 to 29 years and 1.23 (1.07 to 1.15) for ≥30 years across all studies.

We identified 15 studies (10 case-control and five cohort) that assessed the association between prematurity and breast cancer risk (Table 5). Most studies did not observe a significant relationship [13-16,20,25,29,31,33,40,42,45]. Two studies found that extreme prematurity was associated with an increased risk (OR 3.96 [95% CI 1.46 to 10.81] for ≤32 weeks relative to ≥33 weeks [32], and SIR (standardized incidence ratio) 6.7 [95% CI 1.4 to 19.5] for <31 weeks [62]). In contrast, another study [34] found that longer gestation was associated with a significantly increased risk (OR 8.4 [95% CI 1.3 to 54.4] for ≥40 weeks relative to ≤32 weeks).

**Table 5:** Table 5 Studies assessing the association of premature birth and the risk of breast cancer

Type of study	Author	Year	Design	Cases	Controls (or cohort)	Country/place of study	Gestational age (weeks)	OR (95% CI)	Comments
Case-control studies	[42]^a^	1988	PCC	153	461	USA	25–32	1.16 (0.50–1.54)	Matched analysis
							33–40	Referent	
	[13]^b^	1996	NCC	571	1,525	USA	Categorical		Adjusted for age
							40	Referent	
							38–39	0.76 (0.44–1.32)	
							36–37	0.96 (0.59–1.56)	
							Binomial		
							≥ 37	Referent	
							≤ 36	0.82 (0.37–1.82)	
	[14]	1996	PCC	1123	1371	USA	Nonpreterm	Referent	Adjusted for age, menopausal status, and maternal smoking
							Preterm	1.1 (0.5–2.1)	
	[32]^a^	1997	NCC	1,010	2,625	Sweden	≥ 33	Referent	Adjusted for maternal age, matermal socioeconomic status, maternal parity, maternal pre-eclampsia or eclampsia, neonatal jaundice, severe prematurity, twin, and birth weight
							≤ 32	3.96 (1.46–10.81)	
	[33]^b^	1998	PCC	502	433	USA	≥ 43	1.5 (0.8–2.6)	Crude ORs
							37–42	Referent	
							≤ 36	0.9 (0.5–1.8)	
	[15]^a,b^	2000	LCC	480	2,854	USA	≥ 37	Referent	Crude ORs
							33–36	1.34 (0.85–2.13)	
							≤ 32	0.55 (0.19–1.57)	
	[34]^a,b^	2001	LCC	87	87	Sweden	≥ 40	8.4 (1.3–54.4)	Matched analysis by conditional logistic regression
							37–40	3.4 (0.7–17.0)	
							33–36	3.5 (0.7–17.5)	
							≤ 32	Referent	
	[25]^a^	2003	LCohort	127	(1,483)	Sweden	≥ 33	1.08 (0.64–1.70)	Standardized incidence ratio (expected/observed)
							≤ 32	0.92 (0.57–1.41)	
	[45]^a,b^	2004	LCC	2,471	9,801	USA	≥ 37	Referent	Adjusted for age and maternal age at first birth
							32–36	0.91 (0.72–1.13)	
							≤ 31	1.43 (0.90–2.28)	
	[20]^b^	2005	PCC	1,424	1,457	Poland	≥ 37	Referent	Adjusted for age, education, age at menarche, menopausal status and age at menopause, age at first full-term pregnancy, number of full-term pregnancies, family history of breast cancer among first-degree relatives, mammography screening, and current body mass index
							≤ 36	1.01 (0.75–1.32)	
Cohort studies	[62]^c^	2000	LCohort	12	273	Sweden	35	0.2 (0.01–1.3)	Standardized incidence ratio
							33–34	0.7 (0.1–2.0)	
							31–32	2.3 (0.7–5.3)	
							<31	6.7 (1.4–19.5)	
	[16]^c^	2001	LTCCS	2,265	9,060	Sweden	33–36	Referent	Crude ORs
							37–38	1.8 (0.83–4.0)	
							40–44	2.0 (0.88–4.6)	
	[29]^c^	2005	LCohort	367	5,346	Sweden	1 week increase	<50 years	
								0.94 (0.83–1.07)	
	[40]^c^	2006	Cohort	97	5,847	USA	<39	0.77 (0.42–1.4)	Adjusted for age
							39	1.38 (0.78–2.4)	
							40	Referent	
							41+	1.33 (0.67–2.6)	
	[31]^b^	2006	Cohort	249	1024	USA	≥ 43	0.7 (0.2–2.7)	Adjusted for: age at diagnosis, diagnosis year, stage at diagnosis, and birth order, with exception of birth order, which is adjusted for maternal age
							37–42	Referent	
							<37	1.4 (0.7–2.9)	
								*P *for trend = 0.3	

Cohort, cohort study; LCC, case-control study with linkage with population and cancer registry data; LTCCS, twin case-control study by using linkage with birth and cancer registry data; NCC, nested case-control study in cohort; PCC, population-based case-control study. ^a^We included this study in the meta-analysis with categories of ≥33 versus ≤32 months (reference). ^b^We included this study in the meta-analysis with categories of ≥37 versus ≤36 months (reference). ^c^We did not include these studies in the meta-analysis because they employed different categories or a continuous scale, or they did not provide the numbers of cases and controls in the report.

**Figure 6:**
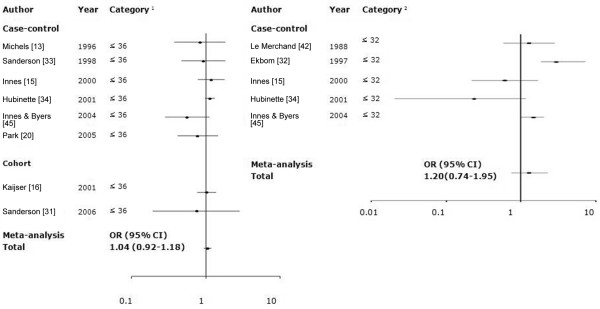
Meta-analysis of studies assessing the association of prematurity and risk for breast cancer. The tests for homogeneity and for publication bias in the studies analyzed are as follows. Category 36+: Q = 5.91 (7 degrees of freedom), *P *= 0.55; Begg test, *P *= 0.11; Egger test, *P *= 0.03. Category 32+: Q = 10.10 (4 degrees of freedom), *P *= 0.04; Begg test, *P *= 0.09; Egger test, *P *= 0.40. ^1^Category of prematurity (week): ≤36 versus ≥37 (reference). ^2^Category of prematurity (week): ≤32 versus ≥33 (reference). CI, confidence interval; OR, odds ratio.

**Figure 7:**
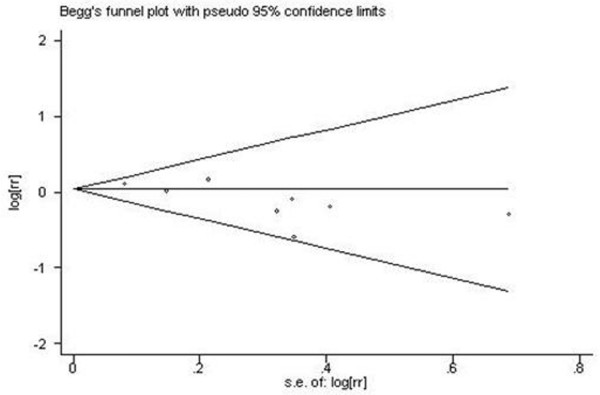
Begg's funnel plot for publication bias in meta-analysis of premature birth and breast cancer risk. Premature birth (gestational age ≤36 weeks) was compared with gestational age ≥37 weeks. Egger test, *P *= 0.03; Begg test, *P *= 0.11. rr, relative risk; s.e., standard error.

There was no significant heterogeneity across studies (*P*-Q test = 0.55), whereas we found no association between prematurity (≤36 weeks) and risk (OR 1.04 [95% CI 0.92 to 1.18]; Figure 6). However, a strong publication bias was observed (*P*-Egger test = 0.03 and *P*-Begg test = 0.11; Figure 7). A significant publication bias occurred because three studies with smaller standard errors of log RR [15,16,34] reported RRs near 1.0, whereas five studies with larger standard errors [13,20,31,33,45] reported substantially reduced RRs. When the analysis was performed for extreme prematurity (≤32 weeks), heterogeneity was also evident across the studies (*P*-Q test = 0.04), and the association was not significant (OR 1.20 [95% CI 0.74 to 1.95]).

**Figure 8:**
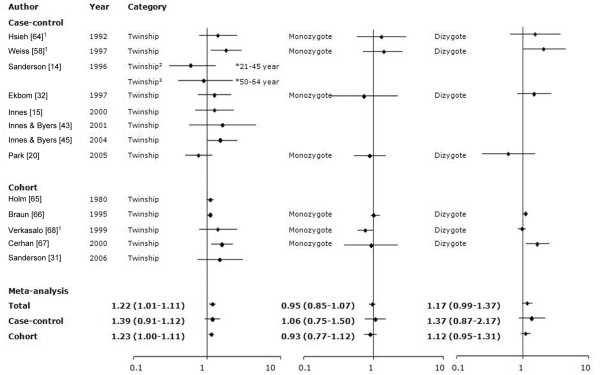
Meta-analysis for the association between twinship and risk for breast cancer. The tests for homogeneity and for publication bias in the studies analyzed are as follows. Twinship: Q = 18.79 (13 degrees of freedom), *P *= 0.13; Begg test, *P *= 0.78; Egger test, *P *= 0.24. Monozygote twin: Q = 5.79 (6 degrees of freedom), *P *= 0.45; Begg test, *P *= 0.55; Egger test, *P *= 0.85. Dizygote twin: Q = 12.53 (6 degrees of freedom), *P *= 0.06; Begg test, *P *= 1.0; Egger test, *P *= 0.3. ^1^The authors used the female twins as the proxy for the monozygote twin and the female twin with male twin as the proxy for the dizygote twin. ^2^Women aged 21 to 45 years. ^3^Women aged 50 to 64 years. CI, confidence interval; OR, odds ratio.

We examined 13 studies (eight case-control and five cohort) that assessed the association between twin status and risk (Table 6). Most studies identified a slightly increased risk among twins [15,31,32,43,45,58,63-66], with five studies demonstrating significant associations [31,58,64-66]. In contrast, some studies observed a slightly reduced risk [14,20,67], with one of the risks being marginally significant [67]. Seven studies [20,32,58,63,65,66] had information on zygosity. Of these studies, two [58,63] used the twins' sex as a proxy for zygosity. For monozygotic twins, a reduction in risk was significant in one study [68]. Most studies failed to observe an association [20,32,58,63,65,66]. Three studies reported a significantly increased risk associated with being a dizygotic twin [58,65,66], whereas other studies reported no association [20,32,63,67] (Figure 8).

**Table 6:** Studies assessing the association of twinship with risk for breast cancer

Type of study	Ref.	Year	Design	Cases	Controls (or cohort)	Country/place of study	Category	OR (95% CI)	Comments
Case-control studies	[63]^a^	1992	MCC	870	2,741	UK, USA	Singleton	Referent	Adjusted for age, study center, parity, age at first birth, age at menarche, height, body mass index, maternal age at birth, birth order, and menopausal status
							Twinship	1.40 (0.77–2.55)	
							Singleton	Referent	
							Monozygote twin	1.30 (0.58–2.92)	
							Dizygote twin	1.54 (0.64–3.71)	
	[14]	1996	PCC	1,134	1,380	USA	Age 21–45		Adjusted for age, menopausal status, and maternal smoking
							Singleton	Referent	
							Twinship	0.6 (0.3–1.3)	
							Age 50–64		
							Singleton	Referent	
							Twinship	0.9 (0.4–2.2)	
	[58]^a^	1997	PCC	2,150	1,961	USA	Singleton	Referent	Adjusted for age, study site, family history of breast cancer, breast biopsy, a combination variable including number of full-term births and age at first full-term pregnancy, age at menarche, menopausal status, body mass index, average lifetime alcohol consumption, and the number of mammograms
							Twinship	1.6 (1.0–2.7)	
							Singleton	Referent	
							Monozygote twin	1.39 (0.7–2.6)	
							Dizygote twin	2.06 (1.0–4.5)	
	[32]	1997	NCC	1,068	2,727	Sweden	Singleton	Referent	Adjusted for maternal age, matermal socioeconomic status, maternal parity, maternal pre-eclampsia or eclampsia, neonatal jaundice, severe prematurity, twin, and birth weight
							Twinship	1.3 (0.8–2.1)	
							Singleton	Referent	
							Monozygote twin	0.7 (0.2–2.2)	
							Dizygote twin	1.5 (0.8–2.7)	
	[15]	2000	LCC	481	2,863	USA	Singleton	Referent	Crude ORs
							Twinship	1.04 (0.51–2.11)	
	[43]	2001	LCC	319	768	USA	Singleton	Referent	Crude ORs
							Twinship	1.6 (0.2–10.1)	
	[45]	2004	LCC	2,522	10,052	USA	Singleton	Referent	Adjusted for age and maternal age at first birth
							Twinship	1.77 (1.05–2.97)	
	[20]	2005	PCC	2,338	2,476	Poland	Singleton	Referent	Adjusted for age, education, age at menarche, menopausal status and age at menopause, age at first full-term pregnancy, number of full-term pregnancy, family history of breast cancer among first-degree relatives, mammography screening, and current body mass index
							Twinship	0.76 (0.49–1.16)	
							Singleton	Referent	
							Monozygote twin	0.90 (0.53–1.52)	
							Dizygote twin	0.58 (0.23–1.47)	

Cohort studies	[64]	1980	LTCohort	270	(16,922)	Denmark	Twinship	1.1 (1.0–1.2)	Observed/expected ratio (95% CI)
	[65]	1995	LTCohort	740	(25,541)	Sweden	Twinship	1.1 (1.0–1.1)	Observed/expected ratio (95% CI)
							Monozygote twin	1.0 (0.9–1.2)	
							Dizygote twin	1.1 (1.0–1.2)	
	[67]	1999	LTCohort	245	(13,176)	Finland	Twinship	0.91 (0.81–1.00)	Observed/expected ratio (95% CI)
							Monozygote twin	0.76 (0.59–0.97)	
							Dizygote twin	0.98 (0.84–1.10)	
	[66]	2000	Cohort	1,230	(29,197)	USA	Singleton	Referent	Adjusted for age, education, family history of breast cancer, age at menarche, age at first birth, height, current body mass index, body mass index at age 18, waist:hip ratio, alcohol drinking, and hormone replacement therapy
							Twinship	1.72 (1.22–2.42)	
							Singleton	Referent	
							Monozygote twin	1.04 (0.43–2.5)	
							Dizygote twin	1.77 (1.16–2.7)	
	[31]	2006	Cohort	249	1,024	USA	Singleton	Referent	Adjusted for age at diagnosis, diagnosis year, stage at diagnosis, and birth order, with exception of birth order, which is adjusted for maternal age
							Twinship	2.5 (1.0–6.2)	

^a^Authors used the female twins as the proxy of the monozygote twin and the female twin with male twin as the proxy of the dizygote twin. Cohort, cohort study; LCC, case-control study with linkage with population and cancer registry data; LTCohort, twin cohort study by using linkage with birth and cancer registry data; MCC, multicenter case-control study; NCC, nested case-control study in cohort; PCC, population-based case-control study.

The Q test for heterogeneity was not significant (*P*-Q test = 0.13), and the meta-analysis of 13 studies examining twin status (without regard to zygosity) found an OR of 1.22 (95% CI 1.01 to 1.11). There was no evidence of any publication bias (*P*-Egger test or *P*-Begg test >0.1). There were little evidence of heterogeneity (*P*-Q test > 0.1 for monozygotic or dizygotic twins), and breast cancer risk was not significantly increased among either monozygotic (OR 0.95 [95% CI 0.85 to 1.07]) or dizygotic (OR 1.17 [95% CI 0.99 to 1.37]) twins, albeit based on limited statistical power. In subgroup analysis by study design, cohort studies identified significantly increased risk (OR 1.23 [95% CI 1.00 to 1.11]) for breast cancer in twins versus singletons, with no study heterogeneity (*P*-Q test = 0.07). Case-control studies showed no association with twin status (OR 1.39 [95% CI 0.91 to 1.12]). There was no evidence of any publication bias (*P*-Egger test or *P*-Begg test > 0.05) among the case-control or cohort studies. In subgroup analysis by study design and zygosity, there were no heterogeneity in studies (*P*-Q test > 0.1). In subgroup analysis by study year, significant heterogeneity by publication year was identified (*P *= 0.01), and the OR (95% CI) for studies published before 2000 was 1.06 (0.97 to 1.47), whereas the OR (95% CI) for studies published in 2000 or later was 1.27 (1.03 to 1.58).

We identified nine studies that assessed the association between maternal or paternal smoking and risk (Table 7). Two cohort studies reported nonsignificantly reduced risks associated with maternal smoking (OR 0.49 [95% CI 0.29 to 1.03] [68]; OR 0.8 [95% CI 0.5 to 1.1] [31]), whereas a case-control study [43] identified a significant positive association (age-adjusted OR 2.7 [95% CI 1.1 to 6.3]), although its crude OR was not statistically significant (OR 1.1 [95% CI 0.7 to 1.7]). The majority of studies, however, identified no associations with maternal [14,20,33,35,58,69] or paternal [20,35,69] smoking during pregnancy.

**Figure 9:**
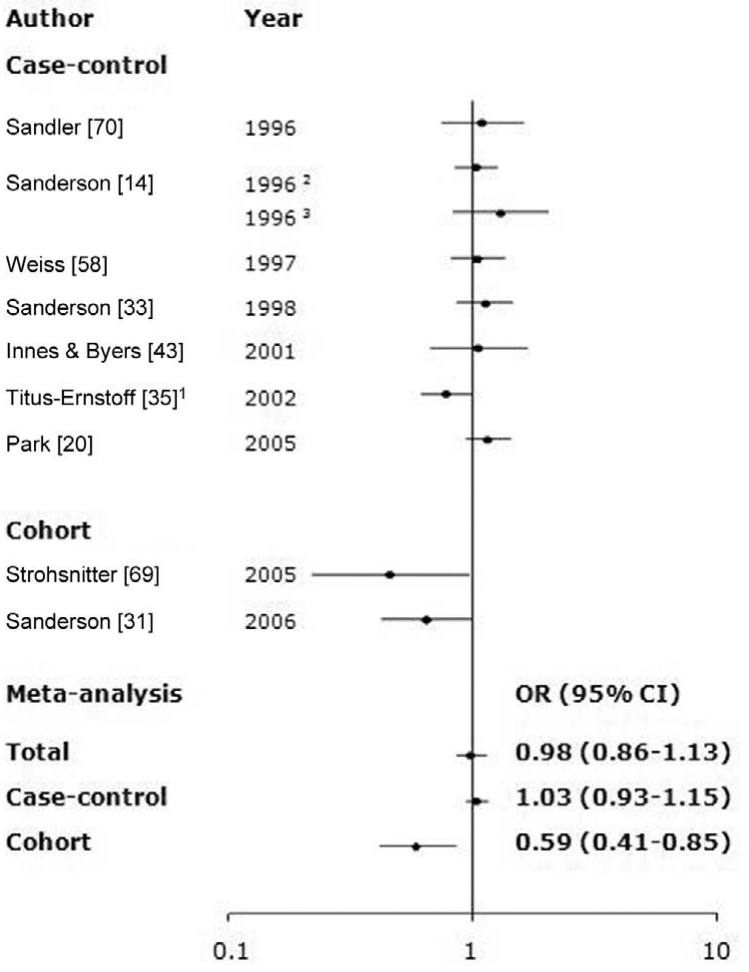
Meta-analysis for the association of maternal smoking during pregnancy with risk for subsequent breast cancer. The tests for homogeneity and for publication bias in the studies analyzed are as follows: Q = 16.90 (9 degrees of freedom), *P *= 0.06; Begg test, *P *= 0.59; Egger test, *P *= 0.31. ^1^Titus-Ernstoff and coworkers [35] classified three categories: nonparental smoking, paternal or maternal smoking only or both parents smoking during pregnancy. The odds ratios (ORs) of father smoking on breast cancer risk was almost unity (OR 1.0, 95% confidence intrval [CI] 0.9 to 1.1). Thus, in this study, the mother smoking and both parents smoking versus nonparental smoking can be considered to the maternal smoking versus no maternal smoking. ^2^Women aged 21 to 45 years. ^3^Women aged 50 to 64 years.

There was no heterogeneity or publication bias (*P*-Q test > 0.05, *P*-Egger test and *P*-Begg test > 0.1 among all studies, case-control or cohort). The meta-analysis for maternal smoking (Figure 9) found no significant association with risk (OR 0.98 [95% CI 0.86 to 1.13]), although cohort studies [40,68] noted a significant negative association with maternal smoking (OR 0.59 [95% CI 0.41 to 0.85]).

**Table 7:** Studies assessing the association of maternal or paternal smoking and the risk of breast cancer

Type of study	Ref.	Year	Design	Cases	Controls (or cohort)	Country/place of study	Smoking status	OR (95% CI)	Comments
Case-control studies	[69]	1996	PCC	53	470	USA	Maternal smoking		Crude ORs
							No	Referent	
							Yes	0.9 (0.4–2.1)	
							Paternal smoking		
							No	Referent	
							Yes	1.3 (0.9–1.7)	
	[14]	1996	PCC	1,086	1,321	USA	Maternal smoking		Adjusted for age, menopausal status, and maternal smoking; OR (95% CI) for maternal smoking among early-onset breast cancer patients (≤ 30 years old) was 1.9 (1.0–3.4)
							Age 21–45 years		
							No	Referent	
							Yes	1.1 (0.9–1.3)	
							Age 50–64 years		
							No	Referent	
							Yes	1.3 (0.9–2.1)	
	[58]	1997	PCC	522	484	USA	Maternal smoking		Adjusted for age, study site, family history of breast cancer, breast biopsy, a combination variable including number of full-term births and age at first full-term pregnancy, age at menarche, menopausal status, body mass index, average lifetime alcohol consumption, and the number of mammograms
							No	Referent	
							Yes	1.1 (0.8–1.4)	
	[33]	1998	PCC	507	433	USA	Maternal smoking		Crude ORs
							No	Referent	
							Yes	1.1 (0.9–1.5)	
	[43]	2001	LCC	319	768	USA	Maternal smoking		Adjusted for attained age
							No	Referent	
							Yes	2.7 (1.1–6.3)	
	[35]^a^	2002	PCC	1,535	1,534	USA	Smoking		Adjusted for age and residential regions (states)
							No	Referent	
							Paternal smoking	1.00 (0.88–1.13)	
							Maternal/parental smoking	1.10 (0.84–1.42)	
	[20]	2005	PCC	2380	2,497	Poland	Maternal smoking		Unadjusted; recalculated
							No	Referent	
							Yes (any exposure)	1.19 (0.97–1.47)	
							Paternal smoking		
							No	Referent	
							Yes (any exposure)	0.90 (0.77–1.05)	
Cohort studies	[31]	2006	Cohort	249	1,024	USA	Maternal smoking	Referent	Adjusted for age at diagnosis, diagnosis year, stage at diagnosis, and birth order, with exception of birth order, which is adjusted for maternal age Crude relative rates
							No	0.8 (0.5–1.1)	
							Yes (any exposure)		
	[68]	2005	Cohort	42	(3,989)	USA	Maternal smoking		
							No	Referent	
							Yes (any exposure)	0.49 (0.29–1.03)	
							≤ 15 cigarettes a day	0.33 (0.12–0.94)	
							>15	0.68 (0.26–1.73)	

^a^Titus-Ernstoff and coworkers [35] classified three categories: nonparental smoking, either paternal or maternal smoking only or both parents smoking during pregnancy. Thus, in this study, the maternal or both parents smoking versus nonparental smoking can be regarded as maternal smoking versus no maternal smoking. Cohort, cohort study; LCC, case-control study by linkage with population data and cancer registry data; PCC, population-based case-control study.

## Discussion

The main finding of our meta-analysis was that heavier birth weight was associated with increased breast cancer risk (18% increased risk for the heaviest weight). Twin status was associated with 1.2-fold higher risk for breast cancer relative to a singleton birth. Although we found some evidence of increased risk associated with older maternal age (OR 1.16 for maternal age ≥30 years), there were heterogeneous findings across study designs.

Most studies identified an increased risk for breast cancer with heavier birth weight, with the association being particularly strong for premenopausal or early-onset breast cancers [14,22,29,30]. Our result was similar to the findings of a recent meta-analysis of 26 studies, which revealed that high birth weight was associated with a RR of 1.23 and restricted to premenopausal women (OR 1.25 [95% CI 1.14 to 1.38) [70]. This analysis grouped birth weight into two categories (classified into high and low birth weight in each study, regardless of specific weight in terms of grams), preventing evaluation of dose-response relationships. We did in fact observe evidence of a dose-response relationship of risk with birth weight, although this was based on a relatively small number of studies involving three or four categories.

Although some studies identified a J-shaped relationship between birth weight and breast cancer risk [12,14,15,18,25,33,35,37,45], others failed to note an increased risk associated with very low birth weights. A recent study involving 3,066 breast cancer patients and 106,504 comparison individuals in a Danish cohort also found no elevated risk among those with very low birth weights [71]. Similarly, our meta-analysis provided little evidence of increased risk for very low birth weights.

Although the mechanisms underlying the association between high birth weight and breast cancer risk remain unclear, it has been suggested that heavier birth weights may result from increased *in utero *exposuresto factors such as insulin-like growth factor-I or estrogens [72-76]. These substances may act as mitogens by increasing the likelihood of genetic mutations [75,77]. However, several studies have failed to find any correlation between umbilical cord estrogen levels and birth weight [78,79]. One study, however, reported a significant positive relationship with estriol [80]. Further studies should be undertaken to assist in the resolution of these conflicting data.

Our analysis found no association of breast cancer risk with birth orders between 2 and 4, but we did note a somewhat reduced risk associated with higher birth orders (at least 5), although the results were heterogeneous across studies. Biologically, pregnancy estrogen levels appear to be higher during first pregnancies and decline in successive pregnancies [81]. Furthermore, cord blood levels of estradiol, estrone, and progesterone are lower for later born than first born children [82]. These findings suggest that the reduced risk associated with higher birth orders may relate to lower estrogen levels. However, evidence supporting birth order as a risk factor for breast cancer is limited, with further investigations needed to evaluate dose-response relationships more fully.

In our meta-analysis, we found some evidence that having been born to an older mother was associated with higher breast cancer risk, although the results were heterogeneous across studies. Our data failed to support the previous studies that suggested a J-shaped relationship between maternal age and breast cancer risk. It was previously suggested that older maternal age may have an adverse effect on the primordial mammary gland of their daughters because of altered hormonal profiles [37] or may linked to the epigenetic change of mtDNA which can lead to breast carcinogenesis by oocyte inheritance [83]. However, the two studies that examined pregnancy estrogen levels according to maternal age found that both total estrogen and estradiol levels were lowest in youngest mothers (<20 years of age), highest in those aged 20 to 24 years, and intermediate in mothers over 25 years of age [78,81]. Thus, it remains unclear from both our meta-analysis as well as from biologic data whether maternal age is a proxy for estrogen or estradiol exposure to fetus. Although it has been suggested that older paternal age may cause germ cell mutations, previous epidemiologic studies have failed to support an association [35,20,69,82,84,85]. Because the purpose of this study was to evaluate whether the intrauterine hormone environment affects subsequent breast cancer risk, our meta-analysis did not include paternal age.

We observed no association between prematurity and breast cancer risk. Biologically, women having abruptio placentae or an extremely premature birth (<32 week) have been shown to have elevated levels of human chorionic gonadotropin and α-fetoprotein, which could inhibit the differentiation of stem cells in human breast tissue cells [15]. Gestational age is related to birth weight, of course, because birth weights in infants born prematurely are lower than those in infants born at term [13].

Twin pregnancies are associated with an approximate doubling of estrogen levels compared with singleton pregnancies [86,87]. Dizygotic twin pregnancies have elevated levels of estrogens and gonadotropins [88-90]. It has therefore been postulated that twins, especially dizygotic twins, could be at an elevated risk for breast cancer. In general, our results did not support differences in risk between monozygotic and dizygotic twins, and there was evidence that risk estimates published after 2000 were qualitatively different from those of earlier studies.

Studies of parental smoking, especially maternal smoking, and daughter's breast cancer risk have yielded inconsistent results. Biologically, maternal smoking, rather than paternal smoking, has a greater impact on the fetus. In the meta-analytic results, both factors failed to exhibit a significant association with risk. Some studies have reported that maternal smoking in pregnancy reduces serum estrogen levels [91,92]. A recent experimental study reported that both estradiol-17β levels and progesterone:estradiol-17β ratios were reduced in pregnant mice exposed to cigarette smoke [93]. However, the relevance of these findings to humans is unclear.

These meta-analyses are based on results from studies involving heterogeneous designs and methodology. We did note between-study heterogeneity for the associations of birth order, maternal age, and twin status. To resolve the heterogeneous findings, we considered the influence of study design and the date of study publication on the results by subgroup analyses. However, heterogeneity in studies could only be explained partially.

Effects of maternal age, birth order, prematurity (cut-off value 32 weeks), and maternal smoking were found to be heterogeneous across study designs, but birth weight and twinning were comparable. Self-reported measures of perinatal factors may be vulnerable to misclassification biases, with differential or nondifferential effects [94,95]. Because studies based on data linkage to medical records have a lower chance of misclassification bias, we conducted subgroup meta-analyses stratified by source of information (data linkage versus self-report) and found no substantial differences in the results. Although the completeness of records is a critical factor in evaluating biases in studies based on data linkage, most papers did not provide details about the completeness of records. We also conducted subgroup meta-analyses stratified by publication year. Only twin status exhibited significant heterogeneity according to publication year (<2000 versus ≥2000).

Our findings may be somewhat inflated because of our dependence on crude rather than adjusted ORs or RRs. A possible misclassification bias for zygosity might have resulted in studies that used sex as a proxy for zygosity [96]. Because this bias would probably attenuate associations, additional investigations are needed to determine the extent of any true association of risk with twin status.

## Conclusion

It has been hypothesized that certain perinatal factors, including birth weight and order, twin pregnancies, prematurity, maternal age, and smoking, may reflect higher estrogenic environments *in utero*, thereby increasing the subsequent risk of breast cancer. Findings of an increase in breast cancer risk among daughters exposed to diethylstilbestrol *in utero *supports this hypothesis [97,98]. Although the current meta-analysis found evidence that higher birth weights are associated with increased breast cancer risk, older maternal age and twin status were less convincingly related, and birth order and prematurity appeared unrelated. Greater birth weights have been attributed to higher maternal estrogens levels, which could affect fetal development [72-74] through epigenetic modifications of breast stem cells [1,99,100]. Although our findings regarding birth weight support the hypothesis that higher estrogen exposures *in utero *may be involved in the subsequent development of breast cancer, further biologic data are needed to elucidate the relationship fully.

## Abbreviations

CI = confidence interval; OR = odds ratio; RR = relative risk.

## Competing interests

The authors declare that they have no competing interests.

## Authors' contributions

SKP collected and selected the all of breast cancer studies, analyzed the data in the study, drafted the manuscript, critically revised the manuscript for important intellectual content, and takes responsibility for the study concept and design, the integrity of the data, and the accuracy of the data analysis. DK participated in design of the study, drafting of the manuscript and interpretation of results, and critically revised the manuscript for important intellectual content. KAM was responsible for the study concept and design, interpreted the findings, and revised the manuscript for important intellectual content. MGC participated in the interpretation of the data and revision of the manuscript. YK was involved in data analysis and revision for important intellectual content. KYY contributed to interpreting the findings and critically revised the manuscript for important intellectual content. LAB led conception and design of the study, the analysis and interpretation of the findings, and revision to the manuscript, and obtained part funding for this research. All authors read and approved the final manuscript.
